# Young People With Type 2 Diabetes Are Under‐Represented in Randomized Clinical Trials

**DOI:** 10.1111/dom.70935

**Published:** 2026-05-28

**Authors:** Bowei Yu, Juliana C. N. Chan, Andrea O. Y. Luk, Hongjiang Wu

**Affiliations:** ^1^ Department of Medicine and Therapeutics The Chinese University of Hong Kong Hong Kong Special Administrative Region People's Republic of China; ^2^ Li Ka Shing Institute of Health Sciences, The Chinese University of Hong Kong Hong Kong Special Administrative Region People's Republic of China; ^3^ Hong Kong Institute of Diabetes and Obesity, The Chinese University of Hong Kong Hong Kong Special Administrative Region People's Republic of China; ^4^ Phase 1 Clinical Trial Centre The Chinese University of Hong Kong Hong Kong Special Administrative Region People's Republic of China

**Keywords:** clinical trial, diabetes complications, randomized trial, type 2 diabetes

Type 2 diabetes is a leading cause of morbidity and premature mortality worldwide. Once considered a disease of older adults, type 2 diabetes is now increasingly affecting younger populations [1]. Young‐onset type 2 diabetes is commonly defined as type 2 diabetes diagnosed at age < 40 years [2], although there is no standard definition. In contrast to the stabilization or decline in the overall incidence of type 2 diabetes in most countries over the past two decades, the incidence of young‐onset type 2 diabetes has risen substantially. Data from the Global Burden of Disease Study have shown that the global age‐standardized incidence of type 2 diabetes among people aged 15–39 years increased by more than 50%, from 117.2 to 183.4 per 100 000 population between 1990 and 2019 [3].

Compared with people with later‐onset type 2 diabetes (defined as type 2 diabetes diagnosed at age ≥ 40 years) and those with type 1 diabetes, people with young‐onset type 2 diabetes have substantially higher lifetime risks of diabetes‐related complications and premature mortality [4]. A meta‐analysis reported that each 1‐year younger age at diagnosis of type 2 diabetes was associated with a 4% higher risk of all‐cause mortality, a 3% higher risk of macrovascular disease and a 5% higher risk of microvascular disease [5]. Despite advances in diabetes care that have significantly reduced hospitalization rates and mortality among people with diabetes worldwide, these improvements have not been observed in young people [6]. Managing type 2 diabetes in young people is challenging because of its aggressive clinical course and substantial psychosocial burden. People diagnosed with type 2 diabetes at a younger age are exposed to glycaemic and cardiometabolic risk for a longer duration across the life course and tend to have a poorer response to glucose‐lowering therapies. They may also face competing educational, occupational and family demands that hinder self‐management and treatment adherence. Socioeconomic disadvantages and psychosocial barriers, including diabetes‐related stigma and self‐blame, may further compromise self‐care and contribute to poorer clinical outcomes [2]. Mental health disorders represent another important but often under‐recognized dimension of the burden of young‐onset type 2 diabetes. Our previous work showed that mental health disorders were the leading contributor to hospitalization burden among young people with type 2 diabetes aged < 40 years [7], accounting for 40% of all hospital bed‐days. The absence of dedicated clinical guidelines for this population further compounds these challenges.

Randomized controlled trials (RCTs) have established the benefits of intensive glycaemic control, intensive blood‐pressure control and statin therapy in people with type 2 diabetes. We reviewed landmark RCTs that have informed major clinical guidelines for glucose, blood pressure and lipid management in type 2 diabetes. Although these trials did not apply eligibility criteria based on age at diabetes diagnosis, they predominantly enrolled middle‐aged and older adults, with very limited representation of younger people at enrollment. In the UK Prospective Diabetes Study (UKPDS), which enrolled adults aged 25–65 years with newly diagnosed type 2 diabetes, fewer than 10% of participants were younger than 40 years at enrollment [8]. In subsequent trials evaluating intensive glucose control in type 2 diabetes, including the Action to Control Cardiovascular Risk in Diabetes (ACCORD) trial [9], the Action in Diabetes and Vascular Disease: Preterax and Diamicron Modified Release Controlled Evaluation (ADVANCE) trial [10], and the Veterans Affairs Diabetes Trial (VADT) [11], the mean age at enrollment ranged from approximately 60–70 years (Figure 1). Data supporting the cardiovascular benefits of intensive blood‐pressure control in type 2 diabetes are derived mainly from the Blood Pressure Control Target in Diabetes (BPROAD) [12] and the Effects of Intensive Systolic Blood Pressure Lowering Treatment in Reducing Risk of Vascular Events (ESPRIT) trials [13]. In both trials, the mean age of participants at enrollment was approximately 65 years. Current clinical guidelines for statin use in diabetes focus primarily on adults aged 40–75 years, with little trial evidence in those younger than 40 years. In a meta‐analysis of 14 statin RCTs, the mean age of participants with diabetes at enrollment was 64 years [14].

**FIGURE 1:**
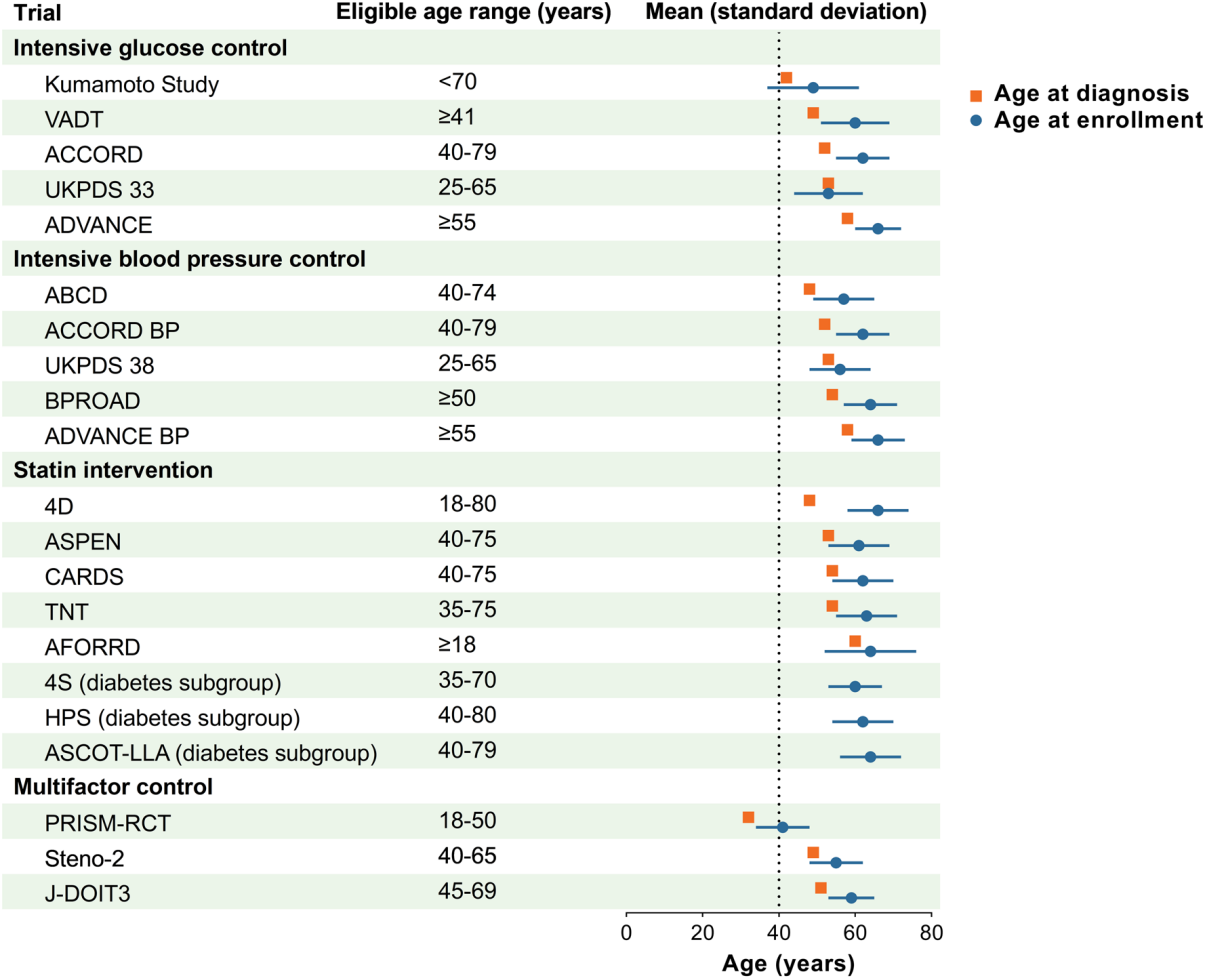
Distribution of age at diabetes diagnosis and age at enrollment in RCTs of intensive glucose control, intensive blood pressure control and statin therapy in people with diabetes. Age at enrollment is presented as the mean with standard deviation. Multifactorial control was defined as simultaneous management of glucose, blood pressure and lipid concentrations. 4D, Die Deutsche Diabetes Dialyse Studie; 4S, Scandinavian Simvastatin Survival Study; ABCD, Appropriate Blood Pressure Control in Diabetes; ACCORD, Action to Control Cardiovascular Risk in Diabetes; ADVANCE, Action in Diabetes and Vascular Disease: Preterax and Diamicron MR Controlled Evaluation; AFORRD, Atorvastatin in Factorial with Omega‐3 EE90 Risk Reduction in Diabetes; ASCOT‐LLA, Anglo‐Scandinavian Cardiac Outcomes Trial–Lipid Lowering Arm; ASPEN, Atorvastatin Study for Prevention of Coronary Heart Disease Endpoints in Non‐Insulin‐Dependent Diabetes Mellitus; BPROAD, Blood Pressure Control Target in Diabetes trial; CARDS, Collaborative Atorvastatin Diabetes Study; HPS, Heart Protection Study; J‐DOIT3, Japan Diabetes Optimal Integrated Treatment Study for 3 Major Risk Factors of Cardiovascular Diseases; PRISM‐RCT, Precision Medicine to Redefine Insulin Secretion and Monogenic Diabetes‐Randomized Controlled Trial; RCT, randomized clinical trial; Steno‐2, Steno type 2 randomized trial; TNT, Treating to New Targets; UKPDS, United Kingdom Prospective Diabetes Study; VADT, Veterans Affairs Diabetes Trial.

Observational studies have reported that suboptimal control of blood glucose, blood pressure and lipid levels is more strongly associated with adverse outcomes in younger than in older people with type 2 diabetes [15]. These findings suggest that earlier and more intensive multifactorial management may yield greater benefit in younger people. However, such findings should be interpreted with caution, as observational studies are inherently subject to several sources of bias, including residual confounding, selection bias (e.g., selective survival in older adults) and reverse causality. These limitations restrict causal inference and make it difficult to draw conclusions about treatment benefit, leaving a major evidence gap in the management of young people with type 2 diabetes.

The growing observational data on the rising burden and poor prognosis of young‐onset type 2 diabetes contrast with the paucity of trial evidence to guide clinical management in this population. We call for dedicated RCTs specifically designed for young people with type 2 diabetes to evaluate treatment and management strategies. Conducting such trials will be challenging because young people with type 2 diabetes represent a relatively small population for recruitment, and long‐term follow‐up will be required to capture sufficient clinical outcome events given their lower short‐term event rates. Future trials should consider more pragmatic designs, including register‐based and adaptive approaches, to enhance feasibility in this population. Linkage to administrative databases or disease registers may facilitate long‐term follow‐up and improve the completeness of outcome ascertainment. Incorporating decentralized elements into clinical trial designs—such as tele‐consultation, wearable sensors, electronic patient‐reported outcome systems and direct‐to‐patient drug delivery—enables the transfer of specific trial‐related activities from fixed research sites and clinics. This approach can reduce the burden of frequent visits and enhance participant enrollment and retention, especially among younger working individuals who encounter difficulties in attending research sites regularly. The Precision Medicine to Redefine Insulin Secretion and Monogenic Diabetes randomized controlled trial (PRISM‐RCT) is a 3‐year trial conducted in Hong Kong Chinese aged 18–50 years with young‐onset diabetes. It showed the benefits of a multicomponent care model with biomarker‐guided classification and algorithm‐based treatment in reducing multiple cardiometabolic risk factors, patient reported outcomes and beta‐cell function in young people with diabetes, and supports the feasibility of conducting structured trials in this understudied population [16, 17].

In conclusion, given the disproportionately high lifetime burden of morbidity and mortality associated with young‐onset type 2 diabetes, urgent efforts are needed to generate trial evidence to guide management in this population. Without such evidence, care for young people with type 2 diabetes will continue to rely on extrapolation from studies conducted largely in middle‐aged and older adults.

## Author Contributions

A.O.Y.L. and H.W. planned the study. B.Y. and H.W. were primarily responsible for drafting and revising the manuscript. J.C.N.C. contributed to the interpretation of results, discussion and revision of the manuscript. All authors had access to and verified the study data. All authors read and approved the final manuscript. H.W. is the guarantor of this work and, as such, had full access to all the data in the study and takes responsibility for the integrity and accuracy of the data.

## Funding

The authors have nothing to report.

## Conflicts of Interest

The authors declare no conflicts of interest.

## Data Availability

Data sharing does not apply to this commentary as no new data were created or analyzed in this study.
